# OpenAltimetry - rapid analysis and visualization of Spaceborne altimeter data

**DOI:** 10.1007/s12145-020-00520-2

**Published:** 2020-09-27

**Authors:** Siri Jodha S. Khalsa, Adrian Borsa, Viswanath Nandigam, Minh Phan, Kai Lin, Christopher Crosby, Helen Fricker, Chaitan Baru, Luis Lopez

**Affiliations:** 1 University of Colorado Boulder, Boulder, CO USA; 2 Scripps Institution of Oceanography, University of California San Diego, La Jolla, CA USA; 3 University of California San Diego, La Jolla, CA USA; 4 UNAVCO, Boulder, CO USA

**Keywords:** Cyberinfrastructure, Data visualization, Data discovery, Altimetry, ICESat-2

## Abstract

NASA’s Ice, Cloud, and land Elevation Satellite-2 (ICESat-2) carries a laser altimeter that fires 10,000 pulses per second towards Earth and records the travel time of individual photons to measure the elevation of the surface below. The volume of data produced by ICESat-2, nearly a TB per day, presents significant challenges for users wishing to efficiently explore the dataset. NASA’s National Snow and Ice Data Center (NSIDC) Distributed Active Archive Center (DAAC), which is responsible for archiving and distributing ICESat-2 data, provides search and subsetting services on mission data products, but providing interactive data discovery and visualization tools needed to assess data coverage and quality in a given area of interest is outside of NSIDC’s mandate. The OpenAltimetry project, a NASA-funded collaboration between NSIDC, UNAVCO and the University of California San Diego, has developed a web-based cyberinfrastructure platform that allows users to locate, visualize, and download ICESat-2 surface elevation data and photon clouds for any location on Earth, on demand. OpenAltimetry also provides access to elevations and waveforms for ICESat (the predecessor mission to ICESat-2). In addition, OpenAltimetry enables data access via APIs, opening opportunities for rapid access, experimentation, and computation via third party applications like Jupyter notebooks. OpenAltimetry emphasizes ease-of-use for new users and rapid access to entire altimetry datasets for experts and has been successful in meeting the needs of different user groups. In this paper we describe the principles that guided the design and development of the OpenAltimetry platform and provide a high-level overview of the cyberinfrastructure components of the system.

## Introduction

The increasing complexity and size of Earth sciences datasets calls for better ways to access, visualize, analyze and understand the data that is being generated. In addition, Earth observation data is increasingly being used to inform decision making regarding pressing environmental and societal issues, driving the need for intuitive data interfaces that support the needs of non-expert users (Kavvada et al. 2020). One of the challenges facing NASA’s Earth Science Data and Information Systems (ESDIS) is that the innovative measurement techniques employed in new Earth observing missions often generate voluminous data products whose utilization is accompanied by steep learning curves and heavy demands on computational resources. NASA’s Advancing Collaborative Connections for Earth System Science (ACCESS) Program is one way that the agency is attempting to address this challenge. ACCESS aims to improve and expand the use of NASA’s Earth science data for scientific research and applications by leveraging modern techniques for discovering, managing and analyzing large and complex Earth science datasets.

This paper describes OpenAltimetry (OA), a cyberinfrastructure platform developed under the ACCESS 2015 program. OA targets a particular category of Earth-observing missions: satellite laser altimeters, and specifically the Ice, Cloud and land Elevation Satellite (ICESat) mission and its successor, ICESat-2. These missions launched in 2003 and in 2018, respectively, and produce data that are fundamentally different from the gridded data products that are generated by most NASA Earth observing satellite missions. Polar orbiting satellite altimeters collect elevation data along densely sampled surface tracks that are widely separated in mid and lower latitudes. These data are only available as a set of file-based hierarchical data products, sequential in time but not spatially organized, with key parameters distributed among multiple data products. Non-expert users must therefore rely on data services provided by the mission and/or archive center, both of which are resource-limited and may not be attuned to the needs of all user constituencies.

The goal of OA is to provide an altimetry-specific data discovery, access and visualization platform that focuses on ease-of-use and transparent high-level functionality, with appeal to new users and experts alike.

## The design drivers for OpenAltimetry

Here we describe the principles and requirements that guided the design of OpenAltimetry. OA was imagined as a cyberinfrastructure tool for easy access to satellite altimeter data from any source. Altimetry data measure surface topography (latitude, longitude, elevation, time), collected along densely-sampled ground tracks which, away from the poles, are separated by distances of km to 10s of km. For missions such as NASA’s ICESat and ICESat-2, which were designed to provide repeated coverage of the same surface locations, altimetry data are not posted at fixed points but are instead stochastically distributed around a predicted ground track (Markus et al. 2017). This presents data visualization and discovery challenges that are not well addressed by current tools for accessing rasterized satellite imagery data.

The initial development of the OA cyberinfrastructure targeted the ICESat dataset (Schutz et al. 2005), which had had undergone its final reprocessing and represented an intermediate step to the more complex and far larger dataset expected from ICESat-2. Existing tools for browsing ICESat data were limited in several ways. NASA’s Reverb client (EOSDIS 2009) allowed users to view the coarse spatial coverage of individual ICESat data files but did not allow users to view representations of data values. The ICESat mission’s internal Science Computing Facility (SCF) visualizer was built for specialist users and prioritized granular control of data selection over visualization functionality or ease of use. NASA’s WorldView (Murphy et al. 2015), which is an excellent example of a user-focused data exploration tool, is nevertheless oriented toward raster data and only shows ICESat/ICESat-2 ground tracks.

We considered the lack of an easy means of traversing the ICESat/ICESat-2 datasets and visualizing their primary observables a missed opportunity for generating interest among new users. More critically, access methods available at the time for ICESat (and planned for ICESat-2) required a high level of familiarity with the data. At the time, few systems existed that were able to serve expert users, who are often the first and most important target of any data distribution system, while still being intuitive enough to accommodate the wider user community.

The OpenTopography^1^ project funded by the US National Science Foundation has demonstrated that online access to data and analysis tools via easy-to-use interfaces can significantly increase data usage across a wide range of users, from students to academic researchers, to those in the commercial sector (Crosby et al. 2020). OA was therefore designed to democratize access to the ICESat and ICESat-2 datasets by leveraging the cyberinfrastructure knowledge and design philosophy behind the OpenTopography data distribution and processing system using state-of-the-art tools and technologies while incorporating feedback from expert users and stakeholders in the development process.

## OpenAltimetry Cyberinfrastructure

The OA cyberinfrastructure is implemented using a service-oriented architecture composed of a presentation tier, a services tier and a data tier. The data tier utilizes a hybrid solution for data management, featuring a highly optimized PostgreSQL database with tiered storage and a decoupled object-based system that stores data in HDF5 file format. The services or application logic tier consist of programs and scripts that process data and are accessible as Web services that can be invoked by applications in the presentation tier. Most users interact with the data via the OA portal in the presentation/applications tier. Several components of this 3-tier architecture are further described below.

The OA cyberinfrastructure was developed in multiple stages, with an initial objective of making ICESat data access seamless and efficient. Since the ICESat mission ended prior to OA development, the final version of the entire dataset (GLAS/ICESat L1B Global Elevation Data, Version 34) was made available in OA. The ICESat-2 dataset (geolocated photon data and surface-specific elevations) presented several additional challenges that required re-architecting and modifying several components across all three tiers, with the biggest impact on the data tier. First, the ICESat-2 mission is more complex than ICESat, simultaneously collecting data along six ground tracks versus ICESat’s single ground track. Second, the ICESat-2 laser fires at 10KHz versus ICESat’s 40 Hz. Finally, ICESat-2 data is being collected continuously through the mission, while ICESat collected data during several month-long campaigns each year. As a result, ICESat-2 is generating several orders of magnitude more data than ICESat, requiring a presentation and data management system that can handle discovery, access and processing of massive volumes of data, as well as a highly streamlined data ingestion workflow.

One of the technical goals from onset was to design a service-oriented architecture that was highly modular, to easily accommodate changing requirements. This enabled rapid prototyping and modular development, lowering the time needed for everything from designing the architecture to deploying changes.

### Cyberinfrastructure design philosophy

OpenAltimetry was built as a web-based application so that end users would not be required to install or download any applications to their local compute environment. In our experience, most users want to quickly browse satellite datasets in an interactive and easy-to-use interface that provides some ability to download data in their area of interest. A majority of users do not require access to all the information contained in the standard data products generated by the mission. However, they need the ability to conveniently acquire the authoritative source product for their spatial and temporal area of interest if needed. Users also require the ability to quickly visualize elevation profiles for the data products including the waveforms (from ICESat) and photon clouds (from ICESat-2).

ICESat data are derived from individual laser shots acquired along specified reference ground tracks, where each track represents the target path of the satellite over a single orbit of earth. There are multiple unique tracks, each of which was traversed multiple times during the mission. ICESat data were temporally organized into campaigns, which are demarcated by start and stop times. Each laser shot is identified by a unique identifier and is described by a set of attributes such as the laser footprint position (latitude/longitude/elevation), the shot time, and the histogram of reflected laser energy (the waveform) that is collected by the instrument. Multiple shots along a track are aggregated into an elevation profile.

ICESat-2 operates similarly to ICESat, however instead of a single laser beam directed toward the ground track, ICESat-2 has six beams organized into three widely spaced (10s km) sets of closely spaced (100 s m) beam pairs. Additionally, ICESat-2 records the time and position of each surface-reflected photon from a laser shot, versus the time vs. energy waveform of ICESat. For ICESat-2, photon data are aggregated into overlapping 40 m track segments, which are somewhat analogous to ICESat footprints. Each segment is identified by a unique identifier and described by a set of attributes (time, position, etc.), similar to that for ICESat. The 10 kHz laser firing rate for ICESat-2 results in photons being returned continuously along the satellite ground track. The aggregate of all collected photons is referred to as the photon cloud, whose shape reveals fine topographic details of the surface.

OA leverages existing open source software projects for efficiency and reduction in development time compared to building software solutions from scratch. Providing alternate pathways (e.g. APIs) to the data was also deemed important, as we had seen with OpenTopography, since it tends to bring an increase in usage of the data and the development of novel applications independent of the portal environment.

While the OA cyberinfrastructure was initially installed and operated on commodity hardware, it was designed to be readily deployable in a cloud environment. That is, it had to have the capability of being migrated to the cloud without major changes or effort in order to potentially leverage the mature commercial cloud offerings including high scalability, load balancing and other optimizations.

Finally, central to OA’s architectural design was the ability to enable continuous ingestion and rapid availability of ICESat-2 data from NASA’s NSIDC DAAC, as well as the ability to add new data products from other missions in the future.

### The OpenAltimetry web portal

Most users of OA interact with the data and visualization features via the application’s portal. The portal’s data discovery interfaces were designed with NASA’s popular EOSDIS Worldview (Murphy et al. 2015) application as a reference in order to provide a recognizable and minimally disruptive user experience. Unlike the NASA EarthData search tool, which must work with hundreds of different datasets within a single user interface, OA takes a more focused approach to data access for a particular class of Earth-orbiting missions: satellite laser altimeters.

In the current implementation of OpenAltimetry, data from the ICESat and ICESat-2 missions have their own map-based data discovery and elevation profile visualization interfaces. This enables us to customize each interface specific to the mission while maintaining a core set of functions that are common and applicable across all missions (e.g. base maps, geographic/polar projection views, etc.). The interactive data discovery user interfaces were custom built using the open source OpenLayers mapping library.^2^ OA uses ESRI ArcGIS World Imagery service for a base layer in the geographic projection view and the MODIS Blue Marble Next Generation layer for the North and South polar projection views. The ICESat and ICESat-2 data are delivered in geographic coordinates. For mid-latitude views on the map interface, we use the geographic projection (EPGS:4326). For polar views, we do projection conversions on the fly using ESRI Web services for the polar projection views: WGS 84 / NSIDC Sea Ice Polar Stereographic North (EPSG:3413) for the north pole and SWEREF99 15 45(EPSG:3031) for the south pole.

OA has implemented the NASA EarthData OAuth module which enables users to login using their EarthData credentials. Once logged in, users are provided additional functions including the capability to download the authoritative and original complete data (subsetted) in HDF5 format from the NSIDC DAAC via their REST API. Users who are logged in can also create and share annotations (referenceable areas of interest with a particular date/campaign selected).

The primary challenge with ICESat and ICESat-2 data is to provide a visual representation of the laser footprints on the map interface without overwhelming the users with huge data volumes. This must be achieved using commodity compute resources, optimized for web-based access. Showing too much data is not useful to the users, overwhelms the client browser, and quickly consumes limited server-side resources.

OA solves this problem by showing a small percentage of data initially and gradually increasing the amount of data with increasing zoom levels on the map interface. The zoom levels, amount of data shown, and functional capabilities vary by mission as well as products within the mission. The amount of data shown at various zoom levels are also pre-determined based on most common client screen resolution data gathered from web analytics. The common capabilities across all interfaces are the rapid response times, availability of visualization tools for plotting elevation data on demand, and ability to quickly download data from a user-selected temporal and spatial area of interest.

### ICESat data discovery interfaces

**Fig. 1:**
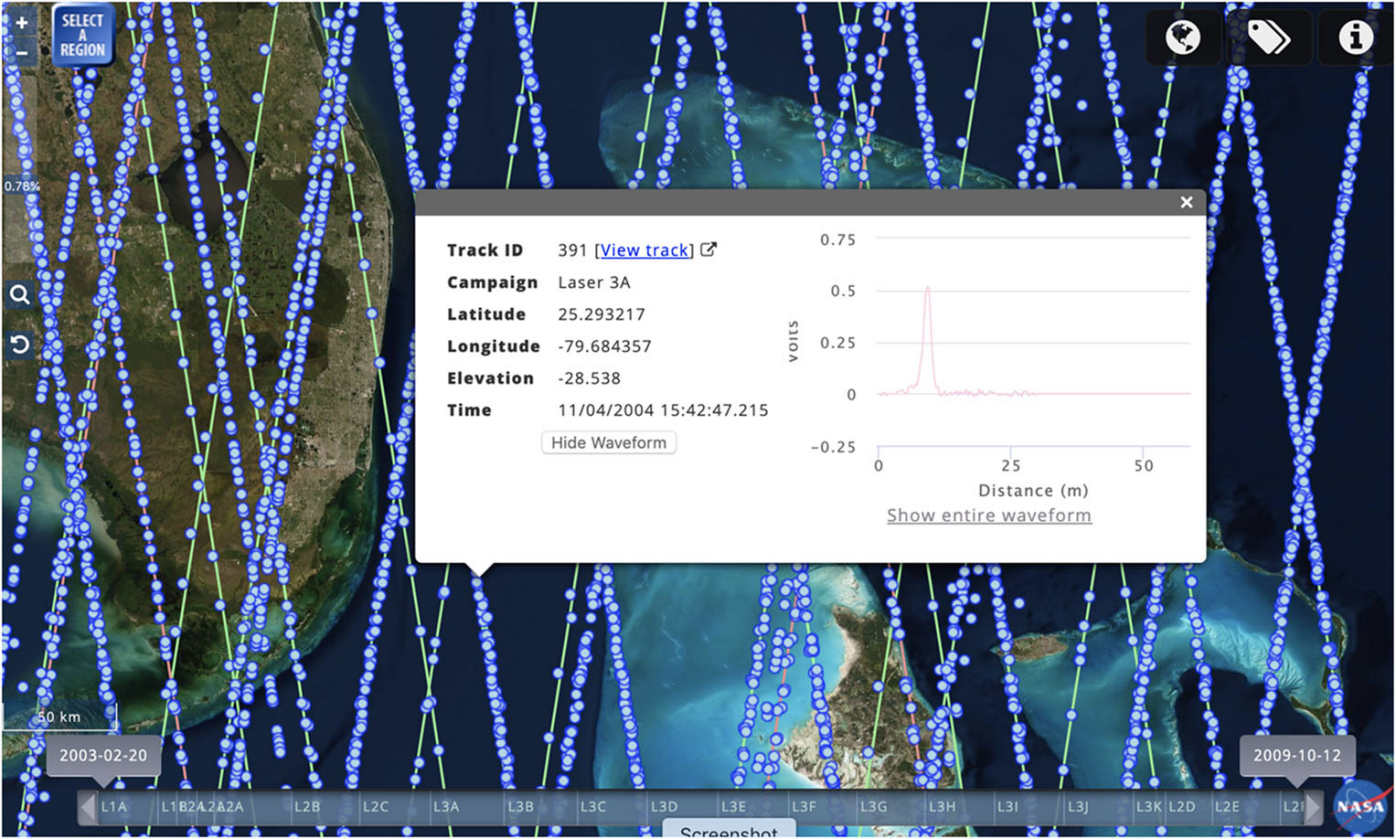
OpenAltimetry user interface showing ICESat reference grounds tracks (green) and location of laser footprints (blue dots). The embedded window displaying information about an individual shot pops up when the user clicks on one of the blue dots

Since the ICESat mission is complete, data for the entire mission is displayed in the map interface. In the ICESat data discovery interface, reference ground tracks are displayed after zooming into a predetermined zoom level, at which point a small percentage of the actual ICESat laser footprints start appearing as blue dots (Fig. 1). This percentage increases as users zoom in to their area of interest, eventually displaying all of the actual footprints. The zoom slider (upper-left of Fig. 1) displays the actual percentage of data being shown.

**Fig. 2:**
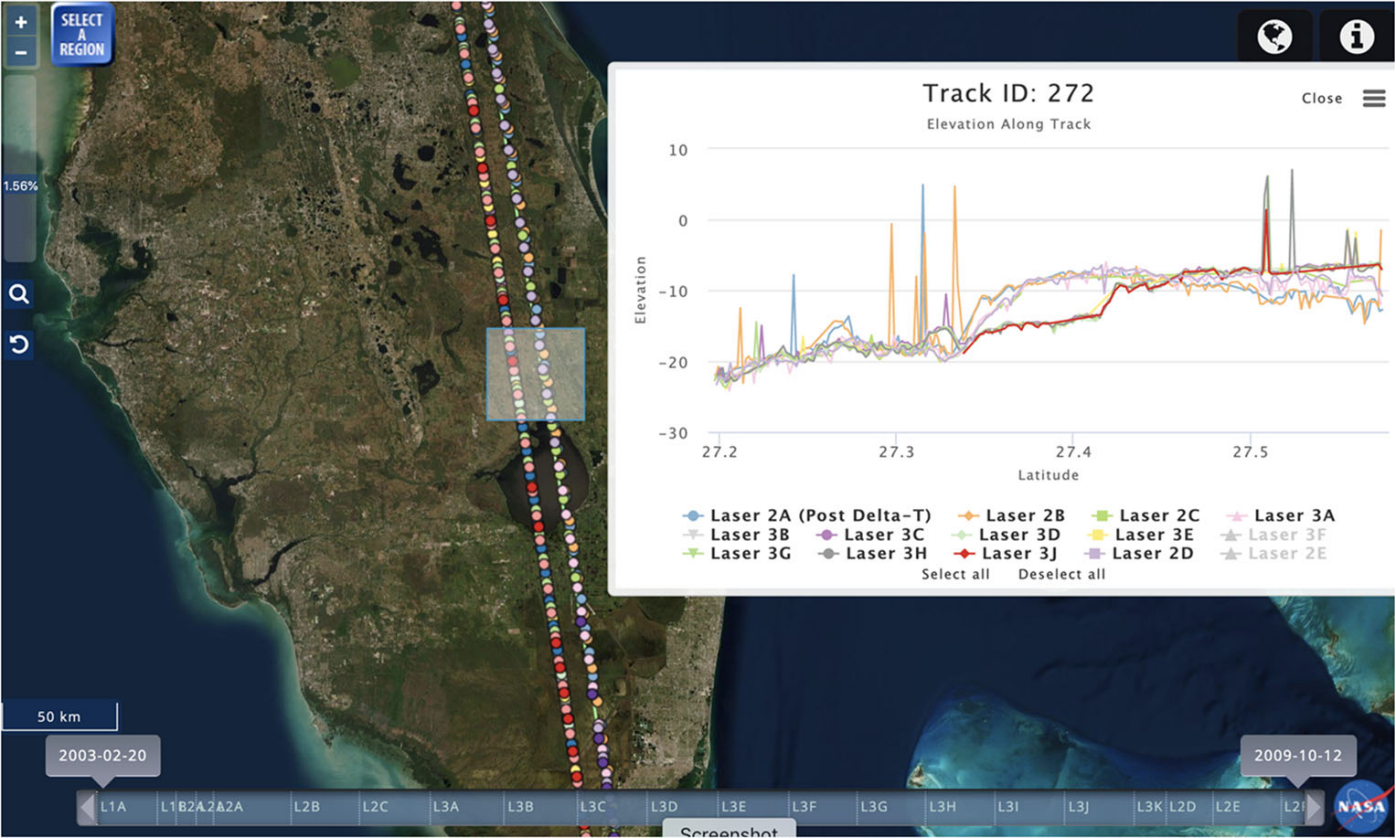
OpenAltimetry display of ICESat laser shots along a single reference ground track, along with an elevation profile derived from all campaigns within the highlighted region. Campaigns are labeled at the bottom of the plot, and can be individually turned on and off in the display. Time slider at the bottom of the window allows restriction of data displayed by campaign

Footprints can be filtered on the fly by reference tracks or by ICESat observation periods (aka laser campaigns; Borsa et al. 2019). At sufficiently high zoom levels, users can draw a bounding box and download basic data attributes (latitude, longitude, elevation, etc.) from the boxed area in comma-separated-value (CSV) format. For levels of zoom that display 100% of the data, the dots corresponding to the footprints are color-coded by campaign (Fig. 2).

Users have the ability to view individual tracks and their laser footprints, which are color coded to represent their campaigns (Fig. 2). This is useful because the actual tracks of laser footprints do not all fall precisely on the reference ground track due to the imprecisions in the orientation of the spacecraft. Users also have the ability to click on individual footprints to bring up a window with additional footprint metadata, including track ID, laser footprint position (latitude/longitude), surface elevation, campaign and shot time (Fig. 1). This window also shows the energy waveform (i.e. the profile of returned energy) for the footprint.

Finally, when users elect to view data from an individual track, they have the ability to select a region and view the elevation profile for a single or multiple campaigns for the track (Fig. 2). Additional options are also available for viewing combined and individual waveforms of the laser footprints, along with the ability to download presentation-quality plots as well as the underlying data. For all the on-the-fly visualizations and plots in OA, we use the open source (non-commercial use) Highcharts Javascript,^3^ an SVG-based multi-platform charting library.

### ICESat-2 data discovery interfaces

**Fig. 3:**
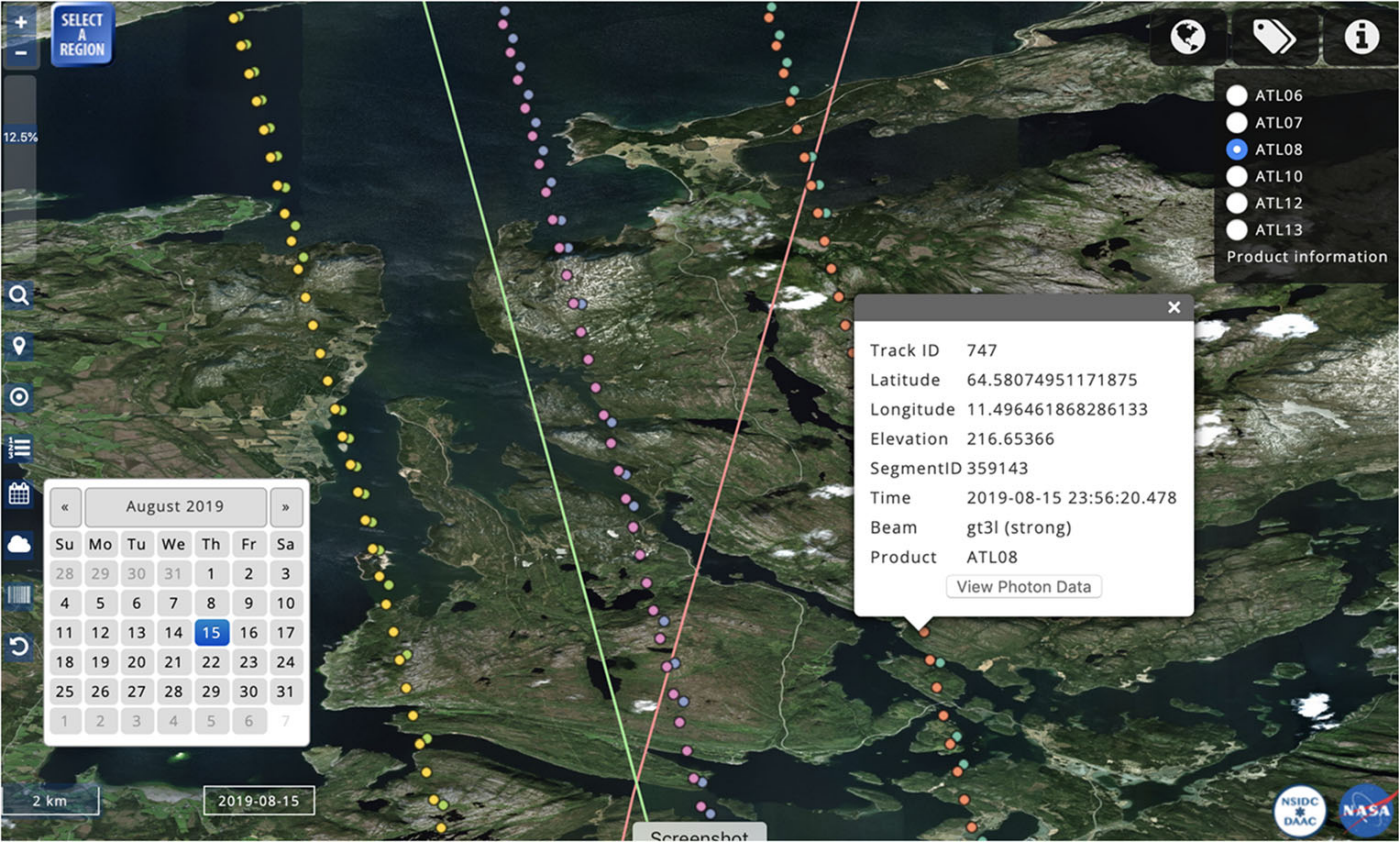
OpenAltimetry display of ICESat-2 laser footprints along a reference ground track (green line). Each of the six laser beams appear in different colors. When a user clicks on a footprint a window appears displaying information about that shot. The pink line shows a different reference ground track

The ICESat-2 data discovery interface has a map-based interface similar to that for ICESat (Fig. 3). A small percentage of ICESat-2 segments are shown initially, increasing to 100% as the user zooms into an area of interest. ICESat-2 segments shown in OA vary with the data product from which they are derived. Segment elevations are provided for land ice height (the ATL06 product), sea ice height (ATL07), land and vegetation heights (ATL08), sea ice freeboard (ATL10), ocean surface height (ATL12) and inland water body heights (ATL13). The segments in these datasets have different spatial coverage and can yield different elevations. Documentation on all ICESat and ICESat-2 standard products are available from NSIDC.^4^ Additionally, elevations are provided for the geolocated photons (ATL03) that serve as the raw data for the other data products.

**Fig. 4:**
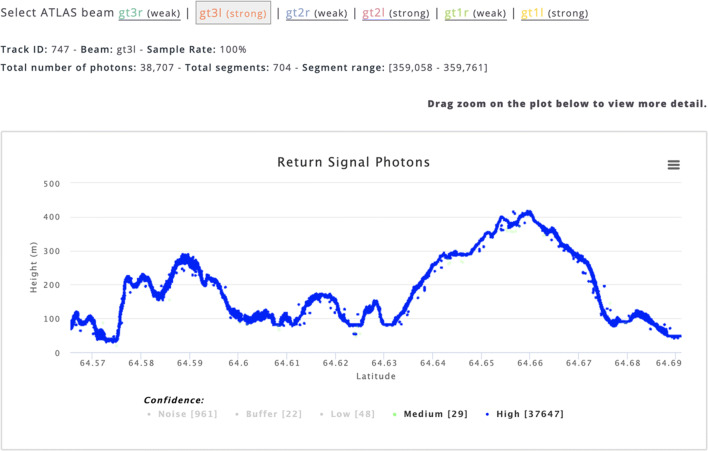
Display of photon elevations from ICESat-2 for one beam along track 747. Points are color coded based on confidence level. High and medium photons are displayed by default but a user can select lower confidence levels by clicking on the labels

Since the ICESat-2 mission is ongoing, with high volumes of data being continuously collected, we display ICESat-2 data in one-day increments in the map interface. When users initially access the ICESat-2 user interface, data from the latest available date is shown for a default data product, which is currently ATL08. Users can select other data products at the top-right of the OA window, or other dates using the calendar in the sidebar (Fig. 3). There are also controls for selecting particular tracks and individual beams. When a user requests a plot of the elevations from a surface-specific product, they are also given the option of viewing the photon data from which the product was derived (Fig. 4).

The percentage of total segments shown at different zoom levels is pre-determined based on performance testing that considers the most popular screen resolutions from web analytics data. It also varies by data product, since segment spacing can vary by product. Users have the ability to click on individual segments to reveal additional metadata, including its position (latitude/longitude/elevation), ID, beam number and track number. Users also have the ability to visualize the corresponding photon data on the fly for that segment (photon height vs latitude) (Fig. 4).

At a particular zoom level, users have the ability to draw a bounding box and view elevation profiles within the selected area. This opens up a new browser window that displays the elevation profiles of the selected data products within that area. In case of ATL08 canopy heights are also displayed and in the case of ATL10 sea ice freeboard is displayed in addition to ice surface elevation. Plots of the return signal photons (Fig. 4) are also available in the new browser window. Since the number of photons can be extremely large, only a sampling of the photons is typically displayed. Users can optionally request to visualize all photon data.

Similar to the case with the ICESat interface, users have the ability to download data as comma-separated values (CSV) for any of the available mission data products, subsetted by the spatial and temporal area of interest. If logged in using EarthData login, users are allowed to download the original HDF5 file from NSIDC DAAC via its REST API, subsetted to the user’s area of interest.

Another powerful capability of the OA portal is the ability for users to add annotations to the datasets. Users can tag specific data views (spatial and temporal) as a persistent URL for easy sharing and collaboration. Annotations also provide the capability to leverage community input in identifying areas of interest and anomalies in the data. Finally, we have also integrated MODIS daily true-color surface product from NASA GIBS (Murphy et al. 2015) to allow users to see cloud and surface conditions for the day of any ICESat-2 acquisition. Users simply toggle a satellite imagery basemap from a button in the sidebar, eliminating the need to access NASA Worldview to view these images.

Capture of user metrics and usage analytics are a key component of OA and have been built into the system from the very onset. These include details on spatial queries performed, data downloads, the number of elevation and photon visualization plots generated, including breakouts by individual products, the number of callbacks to the original HDF5 datasets at NSIDC and spatiotemporal “hot zones” of high data usage, among others. User metrics and usage analytics are vital not just for architecture design and optimization but also as a measurement of success.

### OA data management system

OA utilizes a hybrid solution for data management with a highly optimized PostgreSQL database featuring PostGIS for geographic objects support on tiered storage and a decoupled object-based storage system for storing HDF5 files. The ICESat waveform energy data files and ICESat-2 photon data files are stored in their original HDF5 format. We ingest only the few data elements from the original ICESat-2 HDF5 files needed for OA visualization. This dramatically shortens the data ingestion process, enabling rapid turnaround of newly available data. An added advantage of decoupling the object storage is the ease of porting this data management system to cloud distributed resources for scalability.

The core software component of OA that makes this storage strategy possible is the open source JHDF5 (HDF5 for Java), a Java binding for HDF5,^5^ that extracts waveform and photon data from the HDF5 files on the fly. JHDF5 gives us the capability to fetch specific blocks of the data based on their index, unlike most other libraries (e.g. HDFQL, official HDF5 java), which require loading entire data into the memory and then extracting the needed data. This significantly improves memory usage and performance, vital for rapid response times in the application.

In order to further speed up response times for low zoom levels, we extract a decimated subset of the data products and place them into the PostgreSQL database. Sampling rates vary by product density, ranging from 1 in 2048 for ATL06 and ALT08 to 1 in 128 for ATL12. This eliminates the need to read multiple HDF5 files for showing data footprint locations at global zoom levels. OA performance is thereby improved while adding minimal overhead to the data loading process and minimal additional storage space.

### ICESat-2 data pipeline from NSIDC

OA has a data pipeline established with the NSIDC DAAC to ingest the geolocated photon data product (ATL03) and other surface products (ATL06, ATL07, ATL08, ATL10, ATL12, ATL13) as they become available at NSIDC. The data pipeline is implemented by querying the NSIDC API service with the data product, temporal range and subset of data elements required. The NSIDC service endpoint utilizes URS authentication for their EOSDIS Service Interface results, which satisfies NASA’s requirement to collect metrics on all data access and downloads. Once the request is received, the NSIDC services generate subsetted files for download. These data are then transferred and ingested into the OA data management system using several extract-transform-load (ETL) scripts. The data pipeline with NSIDC is further optimized by parallelizing the data pulls using asynchronous mode. This reduces the overall ETL time at least by half (this varies according to the computational demand at NSIDC) by simultaneously downloading different sections of the dataset in parallel.

### The OpenAltimetry API

We have implemented application programming interfaces (APIs) to allow for programmatic access to all data products available in OA. API-based access to OA data products provide a flexible platform for researchers to design complex data access and processing workflows independent of the processing tools available within the OA portal.

APIs in OA are implemented using the open source Jersey RESTful Web Services framework.^6^ The open source Swagger framework is also used for documenting and consuming these RESTful services.^7^ We have also enabled pre-formatted API endpoints for every elevation plot, where users can conveniently copy and paste the API endpoint URL into their application and pull corresponding data with the subsetted spatial and temporal parameters.

**Fig. 5:**
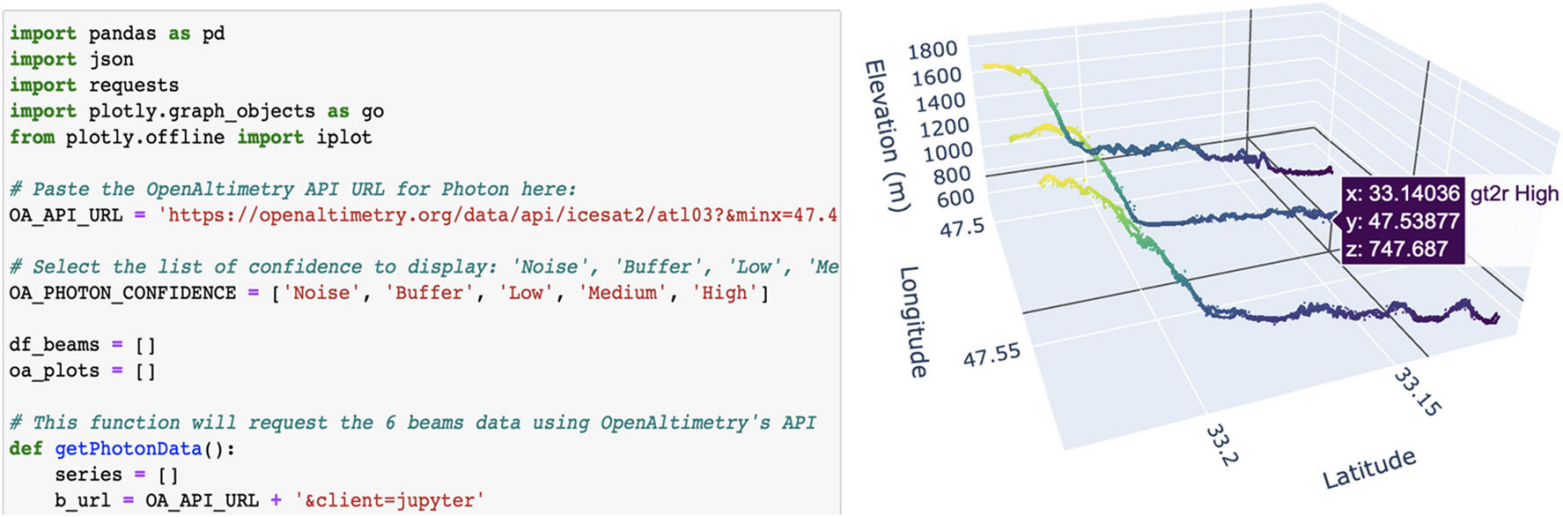
Segment of Jupyter notebook that is linked from the plot pages of OpenAltimetry (left), and a view of the 3-D interactive display of photon elevation data that results when the code is executed

Data outputs are available in multiple formats (including CSV and json) to boost the use of OA data directly in third party applications such as Jupyter notebooks, as well as to promote development of independent algorithms and functionality. To make it more convenient for users, we provide relevant API endpoint URLs under the elevation and photon plots in the user interfaces, as well as links to sample Jupyter Notebooks (Fig. 5) that make use of these endpoints to pull in data.

## Community response and adoption

From its beginning, the OpenAltimetry project had a direct links to the ICESat-2 mission via two of the OA Principal Investigators (PIs), one of whom was also a member of the ICESat-2 science team. Our strategy was to engage with expert data users on the science team from the outset, to understand their needs and to ensure that we were 1) aware of the nuances of the ICESat-2 mission and data products, and 2) informed of major developments impacting the ICESat-2 dataset. The dialogue took place in the form of in-person presentations by the OA PIs to the science team as the interface was being developed for the original ICESat dataset, and occasional updates at science team teleconferences and via email with interested science team members. Since most of the ICESat-2 science team had extensive experience with ICESat, they were able to inform our development efforts even before ICESat-2 was launched. Our development plan was, in fact, predicated on implementing ideas and improvements from an expert cohort before we expanded OA access to the broader community, and this proved to be an effective strategy for building the early version of the OA interface.

One of the initial signs that we were on the right track with this project was the high level of enthusiasm and support among the science team for OA, which has continued up to the present. In parallel with our science team engagement, the OA team and NSIDC staff also conducted outreach with the broader community of non-expert users of ICESat and ICESat-2 data. Our initial efforts took place in partnership with the ICESat-2 Applications Program, wherein OA quickly became the de facto interface for introducing new users to the ICESat-2 dataset due to its rapid data visualization capabilities and its ease of use. Enthusiasm from this different cohort was an independent sign that we were achieving our original design goal to serve users across all levels of expertise. Since the launch of ICESat-2, OA is increasingly featured as a central point of data access for our community. Examples include the use of OA in NSIDC’s presentations at town halls and workshops, in the annual ICESat-2 HackWeek,^8^ and in presentations of PI-led research at scientific conferences and in social media. The AGU 2019 Fall meeting was likely the largest gathering of ICESat-2 data users since the data were publicly released. OA project members and NSIDC DAAC staff all noted unsolicited and repeated references to OA in poster sessions, town hall meetings and other events.

**Fig. 6:**
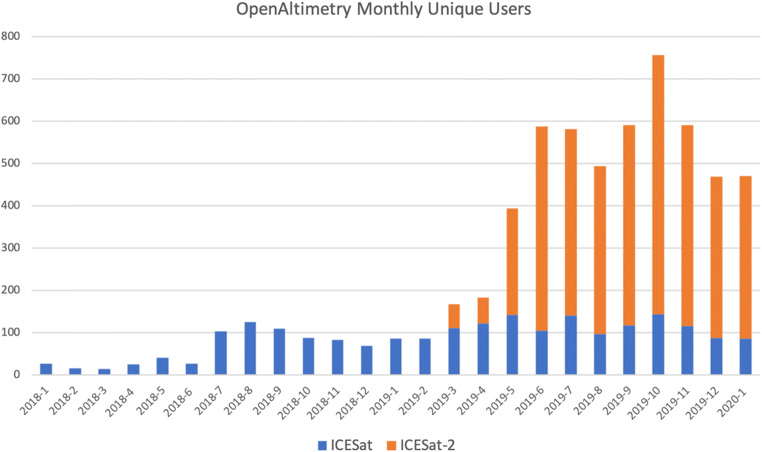
Count of users each month since OpenAltimetry was released, with uses of the ICESat interface in blue and uses of the ICESat-2 interface in orange

An important metric of success for any project is consistent growth in usage. Since its initial deployment, OA has been growing organically, with dramatically elevated usage after ICESat-2 data distribution commenced in March of 2019 (Fig. 6).

## Discussion and conclusions

The most compelling lessons that have been learned through the course of this project are that: 1) novel and complex datasets need tools that provide easy access to and visualization of those data and, more importantly, 2) development must be responsive to user needs and desires from the start. Having the opportunity to demonstrate early versions of OA to the scientists who were developing the ICESat-2 mission and its derived data products was also of great benefit. OpenAltimetry’s success can be attributed to the fact that it enables rapid and responsive exploration at a level of detail in the data that no other tool could provide. This created new use cases and paved the way for new users of the data.

Getting promising technologies into sustained operations is a common challenge. A disturbingly high percentage of cyberinfrastructure projects fail to attract a large enough user community to justify long-term sustainment. A report from the US National Science Foundation (Atkins et al. 2003) suggests that sociological and culture barriers to technology adoption may be a root cause. What is often referred to as the “Valley of Death” (Mcintyre 2014) in business, i.e. the gap between basic research and commercialization of a new product, is, in the context of projects funded with public money, such as NASA’s investments via ACCESS, the gap between having a working prototype and garnering a large enough of a demand for the service it provides that a NASA data center is convinced of the value to its users and is willing to support it in the long term.

OpenAltimetry appears to have succeeded in crossing the Valley of Death. NASA is working to make OpenAltimetry an operational capability within the EOSDIS environment. As a first step we have enabled a seamless handoff from NASA’s EarthData Search Client to OA, whereby a user’s product selection, spatial and temporal bounds and preferred map projection are all passed to OA and the users is able to immediately begin exploring ICESat-2 data in their area of interest.

Based on the response to OpenAltimetry, we expect demand to grow for the inclusion of additional data products. Potential candidates include data products from the Global Ecosystem Dynamics Investigation (GEDI), the Airborne Topographic Mapper (ATM) from Operation IceBridge, and the Land Vegetation and Ice Sensor (LVIS) Facility instrument from NASA Goddard Space Flight Center.

## Data Availability

No new scientific data were generated by the project. Code is open sourced and available at https://github.com/OpenAltimetry

## Footnotes

1 https://opentopography.org/

2 https://opentopography.org

3 https://www.highcharts.com/

4 https://nsidc.org/data/icesat-2

5 https://wiki-bsse.ethz.ch/pages/viewpage.action?pageId=26609113

6 https://eclipse-ee4j.github.io/jersey/

7 https://openaltimetry.org/data/swagger-ui/

8 https://icesat-2hackweek.github.io/
